# Costs of exploratory behavior: the energy trade-off hypothesis and the allocation model tested under caloric restriction

**DOI:** 10.1038/s41598-020-61102-2

**Published:** 2020-03-05

**Authors:** Isaac Peña-Villalobos, Ignacio Casanova-Maldonado, Pablo Lois, Verónica Palma, Pablo Sabat

**Affiliations:** 1 Departamento de Ciencias Ecológicas, Facultad de Ciencias, Universidad de Chile, Santiago, Chile; 2 Laboratorio de Células troncales y Biología del Desarrollo, Departamento de Biología, Facultad de Ciencias, Universidad de Chile, Santiago, Chile; 3 Postgrado Educación, Facultad de Humanidades, Universidad Mayor, Santiago, Chile; 4 Center of Applied Ecology and Sustainability (CAPES), Pontificia Universidad Católica de Chile, Santiago, Chile

**Keywords:** Behavioural ecology, Ecophysiology

## Abstract

In order to maintain the energy balance, animals often exhibit several physiological adjustments when subjected to a decrease in resource availability. Specifically, some rodents show increases in behavioral activity in response to food restriction; a response regarded as a paradox because it would imply an investment in locomotor activity, despite the lack of trophic resources. Here, we aim to explore the possible existence of trade-offs between metabolic variables and behavioral responses when rodents are faced to stochastic deprivation of food and caloric restriction. Adult BALB/c mice were acclimatized for four weeks to four food treatments: two caloric regimens (*ad libitum* and 60% restriction) and two periodicities (continuous and stochastic). In these mice, we analyzed: exploratory behavior and home-cage behavior, basal metabolic rate, citrate synthase and cytochrome oxidase c enzyme activity (in liver and skeletal muscle), body temperature and non-shivering thermogenesis. Our results support the model of allocation, which indicates commitments between metabolic rates and exploratory behavior, in a caloric restricted environment. Specifically, we identify the role of thermogenesis as a pivotal budget item, modulating the reallocation of energy between behavior and basal metabolic rate. We conclude that brown adipose tissue and liver play a key role in the development of paradoxical responses when facing decreased dietary availability.

## Introduction

Due to the finite nature of the energy budget, resource-limited conditions impose changes in the allocation of resources between competing processes, such as growth, reproduction and survival^1,2^. What an animal eats determines the nutrient intake and, ultimately, the distribution of resources between the above mentioned competing biological and physiological processes. Thus, physiological and behavioral traits influencing resource acquisition and use are those having a key effect on the fitness of an individual^3^. In order to maintain the energy balance animals often exhibit physiological adjustments when subjected to a decrease in resource availability. Among those adjustments, both reduction in the basal metabolic rate (BMR) and body temperature (e.g., depression of non-shivering thermogenic activity, NST), allow the resource allocation to other vital processes, in scenarios of energy reductions such as food restriction^4–6^. These responses to food deprivation, documented in various species of marsupial and eutherian mammals^5,7–9^, also entail a reduction of the body mass, metabolic expenditure and a decrease in behavioral activity^10–12^.

Yet, at least three species of rodents (e.g., *Mus musculus, Cricetulus barabensis, Eothenomys miletus*) present a behavioral response considered as “paradoxical” in the face of stochastic deprivation of food or when fed on low-energy diets^13–17^. Indeed, enlargement of the intestine (see ref. in^18^) and changes in behavioral patterns such as an increased locomotion and exploratory activities have been described^4,14,17,19^ and consequently, animals display markedly increased locomotor behavior as well as a decrease in basal metabolic rates and NST^6^. This paradox would imply an investment in structure and function, despite the lack and unpredictable variation of trophic resources. Some of the physiological and molecular mechanisms behind these responses have been identified, whose magnitude appear to be modulated by the endocrine system through plasma variations in the concentrations of leptin and insulin that account for the energy status of the organism^14,20–22^.

Recent studies have evaluated the potential role of energetic constraints as drivers of consistent inter-individual differences in behavior (see^23^ and references therein). The latter is in line with published evidence from food deprivation experiments, claiming that behavioral hyperactivity (shown as wheel running activity or time dedicated to different activities in home-cage behavior) is a function of the severity of food restriction. In this sense, differences among individuals in regards to variability of sources availability have been described, related both to the magnitude and direction of the association between exploratory activity and energy expenditure rates (see^24^ for an example in birds), suggesting that a specific behavioral pattern, could be the result of given environmental conditions (see^25^, for a theoretical approach). Few researchers have addressed, however, the underlying mechanisms behind the relationship between exploratory behaviors and metabolic activity. For example, both features could express some of several relationships, such as trade-offs (e.g. does a limited budget imply a negative relationship between exploratory behaviors and metabolic rates?) or causality (e.g. is there a behavior- dependent modulation of metabolic rates? or vice versa)^24–26^. To date, at least four theoretical models have been proposed to explain the functional association between behavioral traits and rates of energy expenditure^26^. In brief: (i) The allocation model, stating that a negative relationship between the rates of energy expenditure and physical activity is due to the existence of trade-offs in the allocation of resources to both processes; (ii) the performance model, postulating that a positive relationship between the variables emerges because individuals with higher metabolic rates would be able to invest more energy in locomotion activity, or, in other words, metabolic rates could determine the total energy available to an individual; (iii) the substitution model that establishes that in endothermic animals located below the lower critical temperature, the energy cost assigned to the behavior is reduced, and there would be no relationship between the rates of energy expenditure and physical activity. And finally, (iv) the independent model that suggests variation in basic energy requirements neither reduce nor increase the amount of energy available for other processes^23,26,27^.

Several studies support the idea that behavior and metabolism could be associated through mechanistic links. For example, in response to changes in the energy state of the organism, it has been reported that some liver proteins play a role in the modulation of locomotor behavior, such as FGF21 or IGF-I^28,29^. This response is often accompanied by variations in the levels of key metabolic enzymes, such as CS, revealing that its activity may be an indicator of liver function. In addition, skeletal muscles show an increase in their mitochondrial respiration under dietary restriction, which could hypothetically lead to an increment in their performance under a scenario of low food availability^30^.

Summarizing, the current evidence support that in some rodents stochastic food deprivation produces an increase in the frequency exploratory behavior (and/or of locomotion), coupled with a decrease in metabolic rates. Nevertheless, whether a trade-off between energetics and behavior does exist and to what extent this association is dependent on resource availability is less well defined. Thus, a challenging area in the field of behavioral and physiological ecology is the understanding of the causes and ecological consequences of the assumed functional association between maintenance costs and exploratory behavior or activity. This knowledge may shed light on these processes explaining the trade-offs related to the acquisition of energy (i.e., exploration, information and foraging) in different scenarios of resource availability.

The present paper aims to uncover and demonstrate the existence of trade-offs between physiological variables (e.g., BMR, NST, or mitochondrial activity) and behavioral responses when rodents are faced to stochastic deprivation of food and caloric restriction. To this end we developed an experiment, based on the study of the relationship of physiological and behavioral variables, choosing two factors; periodicity in feeding (continuous and stochastic) and caloric content of food (*ad libitum* and 60% of caloric restriction). Adult BALB/c mice were acclimatized for four weeks to these four feeding conditions followed by measurement of the following metabolic features: BMR (measured after treatment), temperature (along all treatment), non-shivering thermogenesis (measured one day after BMR), and mitochondrial activity of liver and skeletal muscle of hind limb legs. Besides, we measured as behavioral variables: the output of open field test or OFT (i.e., movement of individuals, measures before and after treatment) and home-cage behavior (i.e., time expended in to general activity, food, hygiene and rest behaviors, measured after treatment, before BMR measurements). Our results support the model of allocation, which indicates commitments between metabolic rates and exploratory behavior, in a caloric restricted environment.

## Results

### Exploratory behavior and physiological variables

**Figure 1:**
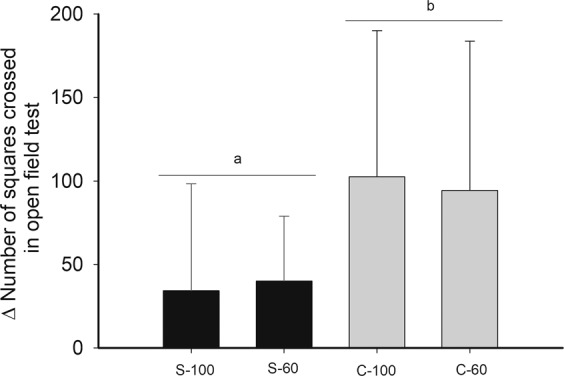
Effect of different food treatments on the trajectory covered in an open field test (number of squares crossed before acclimatization - number of squares crossed after acclimatization) in adult males BALB/c mice.

**Figure 2:**
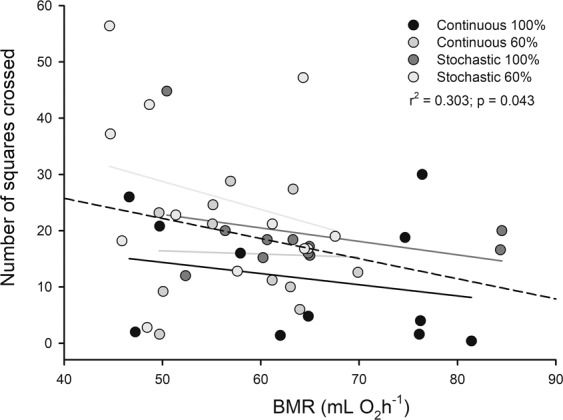
Relationship between basal metabolic rate and the number of squares crossed in an open field test, in adult males BALB/c mice after food treatments as indicated. Dashed line: linear regression for pooled data. There is no statistical significance by treatment.

In order to study behavioral effects of dietary restriction in mice first we examined the repeatability of behavioral traits in the OFT before and after the acclimation period. These calculations indicate that these traits are repeatable and are a good representation of an animal’s behavior. Indeed, repeatability was 0.70 (IC: 0.45–0.84; p < 0.001) for number of squares crossed in the OFT. Besides, Pearson correlation for pooled data was significant for the number of squares crossed in the open field, both after and before acclimation (r^2^ = 0.319; r = 0.565; p < 0.001). Rodents acclimated to the stochastic feeding regime exhibited larger trajectories during the exploration test (~12 meters on average), in comparison with those fed continuously (factorial ANOVA F_(1,39)_ = 0.098, periodicity: p = 0.010, Fig. 1). In addition, there was a trend to form a negative relationship between the caloric intake of rodents and the number of squares crossed in the open field (r^2^ = 0.16, r = −0.400, p = 0.072). In this line, a significant and negative association between the basal metabolic rate and the number of squares crosses was observed (r^2^ = 0.303; p = 0.043, Fig. 2), but not regarding mass-specific BMR.

We found a negative, although weak, relationship between the amount of squares crossed and the liver mass (r^2^ = 0.086, r = −0.293, p = 0.048) and the spleen mass (r^2^ = 0.096, r = −0.309, p = 0.041).

Explanatory models (GLM) of exploratory activity were analyzed based on twenty physiological and anatomical variables and treatments (e.g., liver mass, residuals of liver mass, body mass, basal metabolic rate, minimal temperature, periodicity, etc.). Then, the interaction between these variables was analyzed and finally the best three models were selected. Specifically, the number of squares crossed was highly explained by reduction in the residuals of BAT, and affected by the interaction with periodicity and caloric intake. In a similar fashion, but in less degree, reductions in body mass also explained it. This behavioral variable was affected mainly by periodicity over caloric intake. All statistics are presented in Table 1.

**Table 1:** This table describes the use of the generalized linear model for analyzing the influence of periodicity, caloric intake (as dummy variables), and the interaction between these factors and metabolic rate, temperature and body/organ mass on the exploratory and home-cage behavior.

	Response variable	Models	Variables	Estimate	P	AIC
Open Field Test	Number of squares crossed	Periodicity + Residuals BAT * Body mass	Intercept	7.11	^***^	1835.5
			Periodicity	−0.43	^***^	
			Residuals BAT	−8.26	^***^	
			Body mass	−0.09	^***^	
			Residuals BAT * Body mass	0.31	^***^	
		Periodicity + Caloric intake + Residuals BAT * Body mass	Intercept	7.16	^***^	1826.4
			Periodicity	−0.45	^***^	
			Caloric intake	−0.11	^***^	
			Residuals BAT	−8.79	^***^	
			Body mass	−0.08	^***^	
			Residuals BAT * Body mass	0.32	^***^	
		Periodicity * Caloric intake * Residuals BAT * Body mass	Intercept	10.42	^***^	1335.6
			Periodicity	−7.01	^***^	
			Caloric intake	−6.30	^***^	
			Residuals BAT	−56.48	^***^	
			Body mass	−0.21	^***^	
			Periodicity * Caloric intake	7.77	^***^	
			Caloric intake * Residuals BAT	24.41	^*^	
			Periodicity * Residuals BAT	72.68	^***^	
			Caloric intake *Body mass	0.23	^***^	
			Periodicity * Body mass	0.24	^***^	
			Residuals BAT * Body mass	2.08	^***^	
			Periodicity * Caloric intake* Residuals BAT	−113.91	^***^	
			Periodicity * Caloric intake* Body mass	−0.26	^***^	
			Caloric intake* Residuals BAT* Body mass	1.10	^**^	
			Periodicity *Residuals BAT* Body mass	−2.60	^***^	
			Periodicity *Caloric intake*Residuals BAT* Body mass	4.44	^***^	
Home-cage behavior	Time in general activity (%)	Body mass + mass-specific BMR	Intercept	53.58	^*^	363.89
			Body mass	1.50	^*^	
			Mass-specific BMR	−12.00	^*^	
		Mass-specific BMR + Epididymal WAT	Intercept	85.44	^***^	362.82
			Mass-specific BMR	−11.86	^.^	
			Epididymal WAT	23.19	^*^	
		Epididymal WAT + Minimal temperature	Intercept	100.92	^***^	361.95
			Epididymal WAT	25.96	^*^	
			Minimal temperature	−1.50	.	
	Time resting (%)	Residuals BMR + Residuals liver	Intercept	13.74	^***^	326.49
			Residuals BMR	0.56	^***^	
			Residuals liver	18.39	^*^	
		Periodicity * BMR	Intercept	−39.74	^**^	330.8
			Periodicity	53.10	^**^	
			BMR	0.88	^***^	
			Periodicity * BMR	−0.88	^**^	
		Minimal temperature + Residuals liver	Intercept	−35.45	^*^	327.24
			Minimal temperature	1.78	^**^	
			Residuals liver	19.60	^**^	

All non-significant interaction effects (p > 0.05) in the GLM analyses were removed to obtain the best-fitted model in each case. We choose the best three models by Akaike’s information criterion (AIC). Abbreviation: BAT = Brown adipose tissue, BMR = Basal metabolic rate, WAT = White adipose tissue. Significance codes: 0 ‘***’− 0.001 ‘**’− 0.01‘*’ −0.05 ‘.’.

### Home-cage behavior and physiological variables

**Figure 3:**
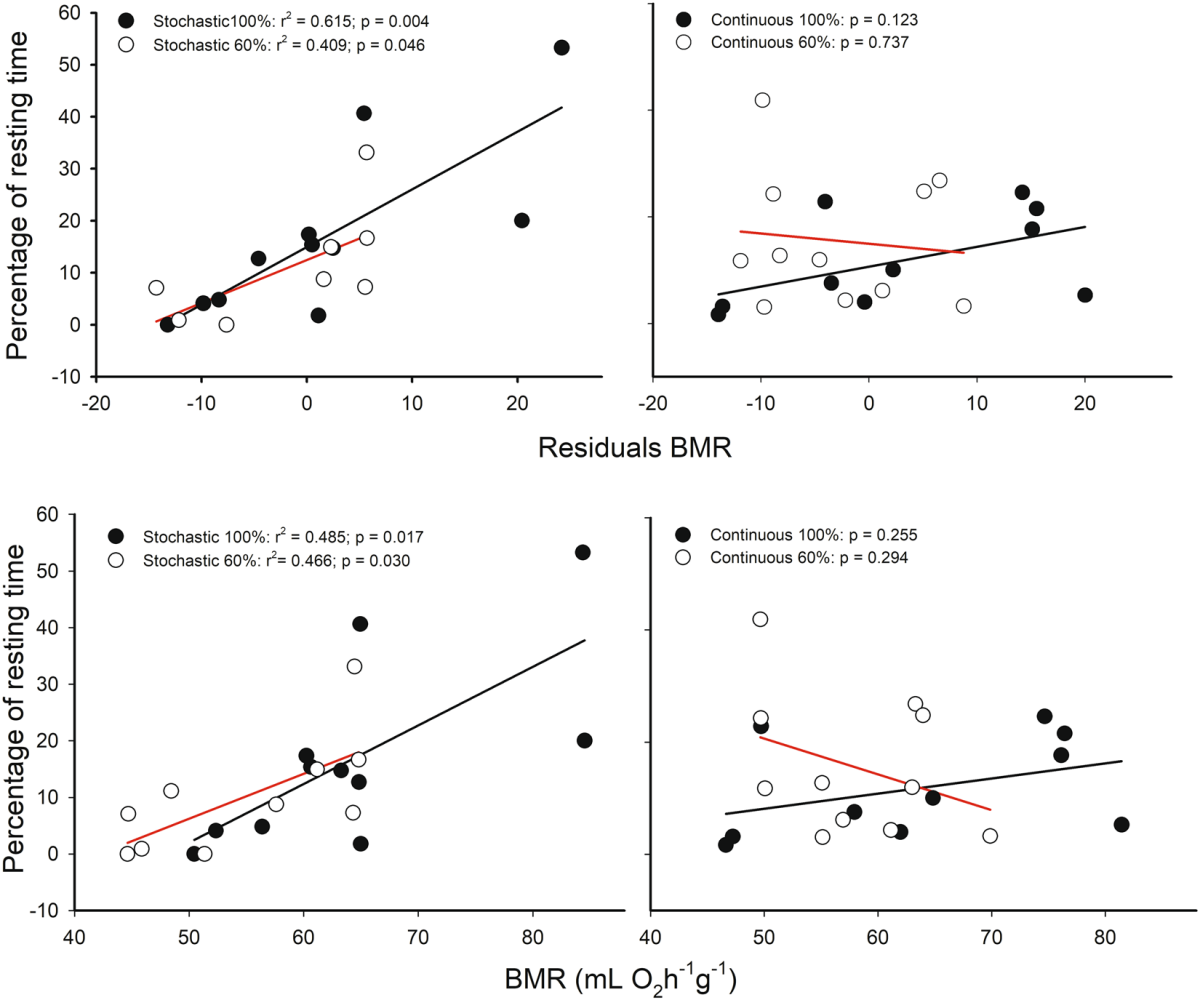
Relationships between energy expenditure rates corrected by body mass or BMR residuals versus body mass and the percentage of rest or inactivity time of *Mus musculus* individuals. Red lines indicate linear regression for mice under caloric restriction at 60%, and black line *ad libitum* feeding.

**Figure 4:**
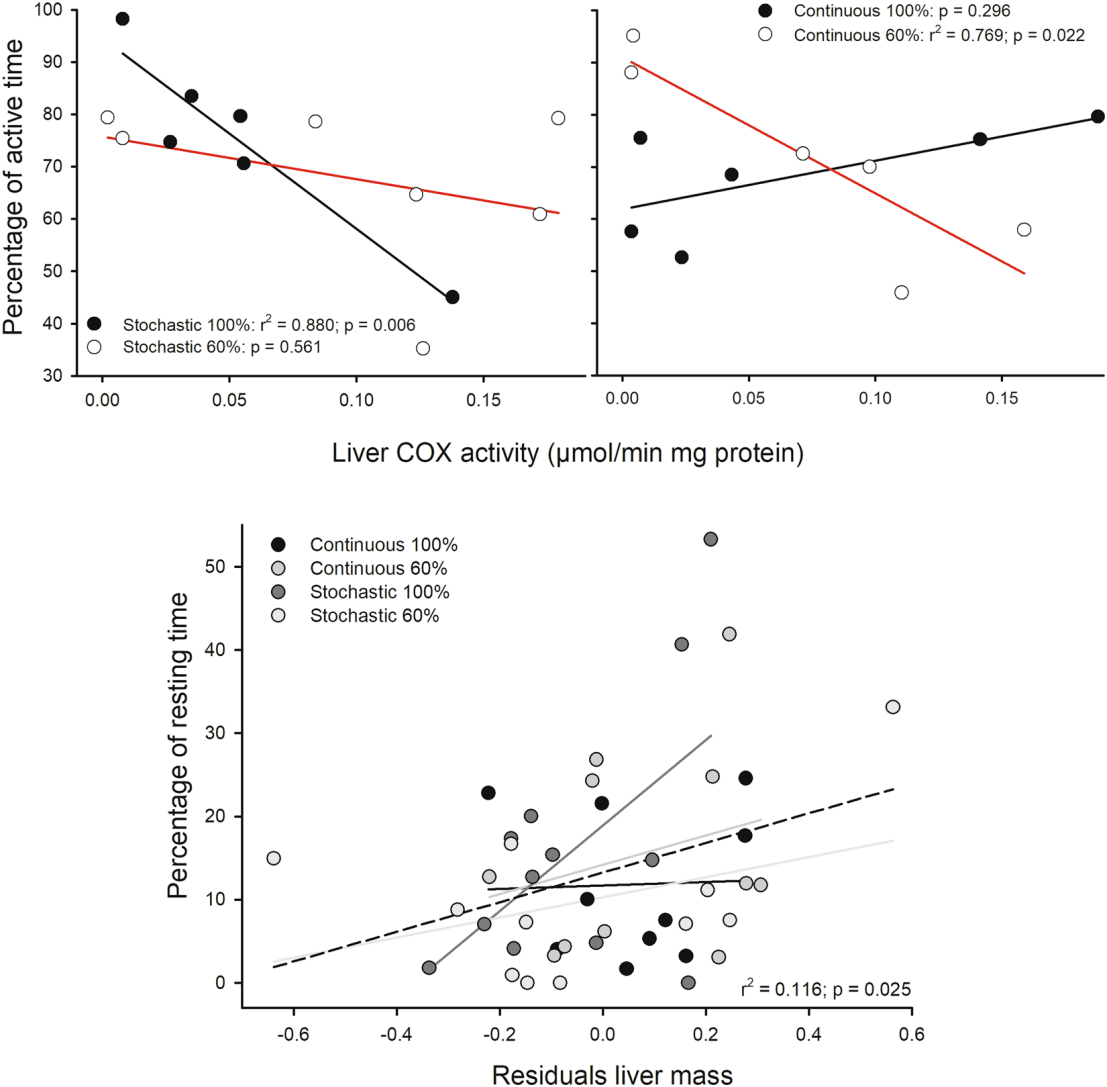
Relationships between liver and home-cage behavior, for mice acclimatized for four weeks to four food treatments: two caloric regimens (*ad libitum* and 60% restriction) and two periodicities (continuous and stochastic). Upper panel show the association between percentages of time spent in locomotor activity and the enzymatic activity of cytochrome c oxidase from liver. Red lines indicate linear regression for mice under caloric restriction at 60%, and black line *ad libitum* feeding. Below, liver residuals against body mass show a positive relation with percentage of time spent in resting. Dashed line: linear regression for pooled data. There is no statistical significance by treatment.

Individuals that allocated a high proportion of time to rest during the activity phase exhibited higher residual BMR, as well as higher mass-specific BMR when analyses were done in pooled data (r^2^ = 0.233, r = 0.483, p = 0.001 and r^2^ = 0.275, r = 0.524, p < 0.001, respectively). However, when this association was evaluated per treatment, only in the stochastic groups these relationship were found to be significant for BMR (r^2^ = 0.466; r = 0.682; p = 0.030 and r^2^ = 0.485; r = 0.696; p = 0.017, in S-60 and S-100 respectively) and for residual BMR (r^2^ = 0.409; r = 0.639; p = 0.046 and r^2^ = 0.615; r = 0.784; p = 0.004, in S-60 and S-100 respectively, Fig. 3). Also, a positive relationship between the percentage of time allocated to rest and residual of the liver mass was found when analyses were done in pooled data (r^2^ = 0.116, r = 0.341, p = 0.025, Fig. 4).

Regarding the time animals spent developing locomotor activity in their home-cages, we found a negative association between mass-specific BMR and percentage of general activity time when analyses were done in pooled data (r^2^ = 0.108; r = −0.329; p = 0.033). But when this association was evaluated per treatment, only in the S-100 group this relationship was found to be significant (r^2^ = 0.573; r = −0.757; p = 0.007). Besides, a negative association between the proportion of time in which animals were found to be active and the hepatic activity of COX, was revealed when analyses were done in pooled data (r^2^ = 0.214, r = −0.462, p = 0.026), Fig. 4. However, when this association was evaluated per treatment, only in S-100 and C-60 groups these relationship were found to be significant (r^2^ = 0.880, r = −0.938, p = 0.006; r^2^ = 0.769, r = −0.877, p = 0.022, respectively), Fig. 4.

As for the CS enzyme in the liver, only a trend in the negative relationship with the percentage of time in activity was observed (r^2^ = 0.163, r = −0.404, p = 0.056).

**Figure 5:**
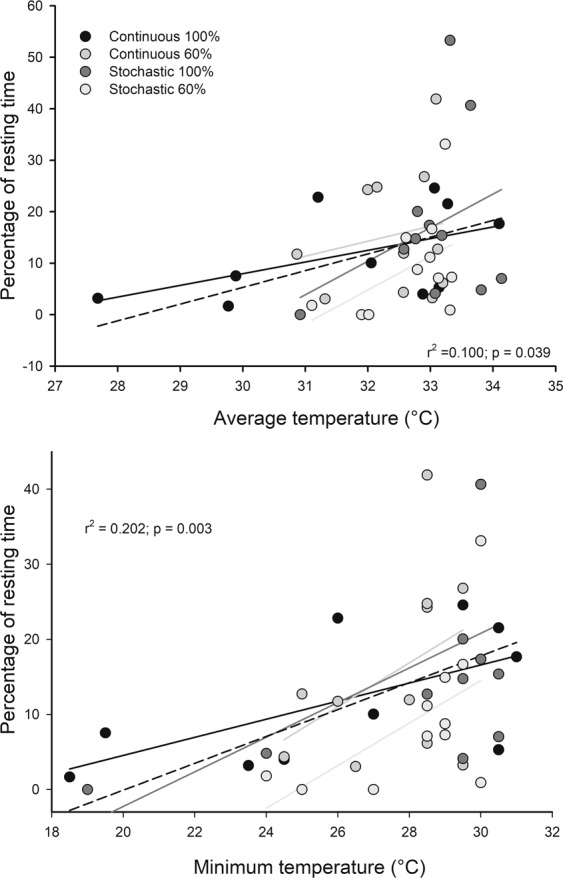
Relationships between surface skin temperatures and the percentage of rest or inactivity time of *Mus musculus* acclimatized for four weeks to four different food treatments. Dashed line: linear regression for pooled data. There is no statistical significance by treatment.

The activity of rodents also was associated with changes in body temperature. In fact, when analyzing the whole data set, individuals with higher minimal and average dorsal skin temperatures, spent most of their time resting (r^2^ = 0.202, r = 0.450, p = 0.003 and r^2^ = 0.100 r = 0.316, p = 0.039, respectively), Fig. 5.

**Figure 6:**
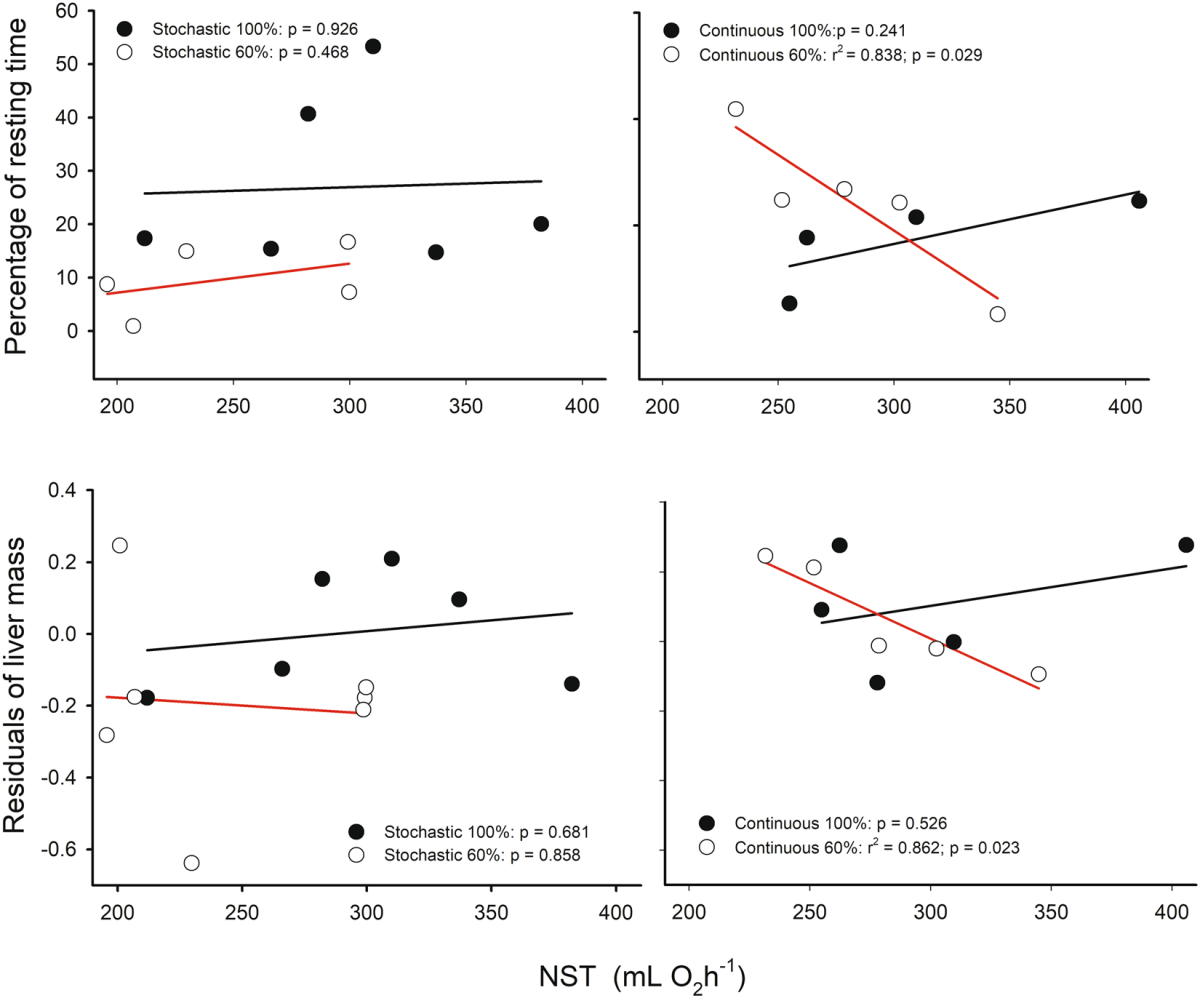
Relationships between home-cage behaviors or liver, and non-shivering thermogenesis, for mice acclimatized for four weeks to four food treatments. Upper panel shows the association between percentages of time spent in resting and the oxygen consumption under norepinephrine stimulation. Below, association between liver residuals against body mass and non-shivering thermogenesis. Red lines indicate linear regression for mice under caloric restriction at 60%, and black line *ad libitum* feeding.

Regarding the origin of thermogenic activity, NST was positively correlated with dorsal skin temperatures of the individuals (r^2^ = 0.128; r = 0.447; p = 0.037). When the analyses were done separating by experimental treatment, only the C-60 group revealed a negative association between NST and residuals of liver mass and time spent resting (r^2^ = 0.862; r = −0.928; p = 0.023 and r^2^ = 0.838; r = −0.915; p = 0.029, respectively), Fig. 6. These results are accompanied by the positive relation between BAT mass and percentage of time in activity found in these group (r^2^ = 0.457; r = 0.676; p = 0.023).

**Figure 7:**
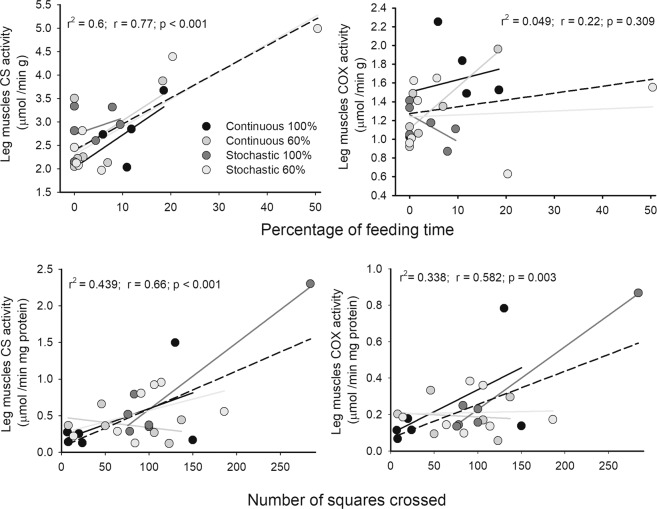
Relationships between mitochondrial enzymes from leg muscles and home-cage or exploratory behavior, for mice acclimatized for four weeks to four food treatments. Upper panel shows the association between percentages of time spent in feeding and the citrate synthase (CS)/cytochrome c oxidase (COX) from leg muscles. Only stochastic 60% treatment shows a significant association for CS activity (r^2^ = 0.840; p = 0.01). Below, show and positive association between CS/COX and squares crossed in open field test. In both analyzes, stochastic 100% group presented a significant association (r^2^ = 0.91; p = 0.003 and r^2^ = 0.97; p < 0.001, respectively). Dashed line: linear regression for pooled data.

A positive relationship was found between the percentage of time devoted to feeding and citrate synthase activity of the hind limbs (r^2^ = 0.600, r = 0.775, p < 0.001). Likewise, individuals that showed a high activity of CS and COX, standardized by gram of proteins of the hind limbs, in turn showed greater displacements on the surface of the open field test (r^2^ = 0.439, r = 0.662, p < 0.001 and r^2^ = 0.338, r = 0.582, p = 0.003, respectively), Fig. 7.

Through GLM models, we found that the time spent in general activity, was related negatively with mass-specific BMR. However, epididymal WAT showed a positive relation with this behavioral feature. Interestingly, the time spent in resting was explained positively, mainly by residuals of liver against body mass. All statistics are presented in Table 1.

## Discussion

The main objective of our study was to evaluate the effect of environmental food stochasticity and availability on selected physiological, biochemical and behavioral traits in a rodent model. Behavioral activity can be described as the levels of general activity of an individual in a familiar situation, while exploration is defined as the response of an individual to novel situations^31^. These behaviors are associated with foraging activity, territorial defense patrols, encounters of breeding pairs and habitat choices^32,33^. Exploration is also defined as the entire collection of seemingly unguided behaviors including movement through space, which is prompted by novel environments^34^. Previous studies suggested that higher activity and exploration capacity would increase the survival of organisms^35^.

In particular, organisms that dwell in unpredictable or unproductive environments will benefit from having an exploratory behavior and low basal metabolic rates since this would increase their probability of finding scarce resources and, hence, survival during frequent resource shortages^36^. In this line, our results support the so-called allocation model^27^. Indeed, we consider that in the present experimental set up (mice under food restriction), the thermogenic activity was a budget item, which was affected by starvation, allowing the allocation of energy from metabolic rate to exploratory behavior.

Interestingly, our results confirm that behavioral repertories seem to respond differentially to environmental signal and conditions^37^. In the aforementioned study the trajectory was affected by periodicity but not by the food availability. In fact, the groups acclimatized to a stochastic feeding, presented a greater Δ_t_ in the exploration test than those acclimated to the continuous regime. Besides, our data reveal that individuals, who traveled the greatest distances, together, presented reductions in the BMR (see Fig. 2). These results strongly support the energy allocation model (See^26,27^), in which a compromise between the rates of energy expenditure and the locomotion activity performed by individuals takes place. These findings are also supported by the analyses of GLM, which revealed a negative relationship between mass-specific BMR and time in general activity. Apparently, this relationship between metabolic rates and behavior will often be determined by genetic constrains but also be modulated by the environment. In this vein, by using an evolutionary experimental approach, some authors concluded that caloric restriction increased more strongly the spontaneous physical activity in laboratory mice divergently selected for low BMR, in comparison with a higher BMR line^38^.

The analysis from home-cage behavior (i.e., their cages) leads to similar conclusions to those that come from the exploration tests. In this sense, it was found that individuals who spent most time in resting behavior had higher metabolic rates, supporting that energy condition caused by the access to resources would determine the amount of energy allocated to energy maintenance and locomotion activity. Regarding this high locomotion activity, despite we do not observe a reduction in the activity, several studies indicate that sustaining high levels of activity under conditions of caloric restriction is not always possible. In fact, there would be biphasic responses, which would involve an increase in locomotive activity, followed by a chronic decrease^39^.

### Role of liver

The results of maintenance energy expenditure rates along with behavioral experiments provide empirical support to the resource allocation model. However, what are the underlying mechanisms or the physiological basis that would be behind this phenomenon in the context of variation of food availability? Of note, it has been show that liver reduces its key metabolic enzyme activities due to the restriction of food intake^40^. Hence, this organ could be a key structure in the generation of the described trade-offs between metabolism and locomotor activity. This conclusion could be supported by evidence, which suggest that caloric restriction influences key metabolic enzyme activities (e.g., citrate synthase)^41^. Indeed, some studies have found that hepatic metabolism could modulate the locomotor behavior and temperature by means of a transcriptional regulation of FGF21 (a growth factor hormone produced mainly in the liver upon fasting and protein restriction) through mTORC1 (an energetic or nutritional sensor)^29^. This hormone might be involved in the adaptive response of body temperature to chronic starvation^42^ and is also implicated in feeding behavior^43^. Besides, there are evidences that suggest the role of liver-derived IGF-I (an anabolic hormone, insulin-like growth factor1) in the regulation of locomotor activity in mice^28^, and in the hypothermic response to caloric restriction^44^. Then, all evidences indicate that several physiological pathways could link liver metabolism, behavior and temperature. In addition, our observation that the liver is as a key structure in the allocation hypothesis is supported by recognizing that the most active individuals in the scotophase have lower COX activities in liver tissue (see Fig. 4). This last observation, regarding catabolic abilities, is valuable in the context of understanding metabolic effects, since COX is an enzyme that directly participates in the reduction of oxygen inside the mitochondria^45^. In addition, this finding is relevant, considering that previous work has only focused on the change of organ masses, and failed to address biochemical adjustments to explain the relationships between metabolism and behavioral traits^46^.

### Role of temperature and NST

From a bioenergetic perspective, the physiological adjustments developed by a caloric restriction would involve an allostatic energy challenge, where “set points” and other boundary of control may change with environmental conditions^47^. In line whit such definition, our data indicate changes in temperature regulation associated to modifications in behavior. Specifically, our results show an inverse relation between temperature variables (i.e., liver catabolism, skin temperature) and locomotor activity (i.e., percentage of activity time). Similar results have been found in multiple studies that address food stochasticity and caloric restriction in rodents^16,48–55^. This reduction of temperature is possible, since rodents are a group of homeotherms capable of lowering their body temperature below 31 °C^15^ and even prefer colder temperatures than their thermoneutral zones^56^. This characteristic presented by rodents, entails their ability to develop thermoregulatory adjustments and suppress energy expenditure rates (see^57^). In addition, it has been proposed that this could be a strategy that allows coping with adverse conditions, maximizing biological adequacy^55^.

Earlier studies have revealed a relation between brown BAT and cold acclimation. Specifically, BAT was identified as a thermogenic organ and shown to be the major site of thermoregulatory NST in rats acclimated to the cold^58,59^. Consequently, we find several negative associations between both BAT mass and NST, with behavioral and hepatic features. Indicating that NST could be compromising liver mass and behavior under caloric restriction. At the same time, more active individuals had lower temperatures, suggesting that a reallocation of energy from mitochondrial decoupling processes and hydrolysis of ATP, towards the development of locomotor activity might occur. In terms of mechanistic, we hypothesize that at tissular level molecular sensors of energy, such as mTOR or sirtuins, could modulate differential processes in the same individual. For example, oxidative activity occurring in on tissue, while anabolism or another energetically expensive process (e.g., locomotor activity) occurring in another^60^. From this “allocation” (or differential response to nutrient availability) an adaptive adjustment to caloric restriction by means of the restricted use of energy and biomolecules can result. The later could explain, at least partially, results obtained in several species of rodents. For instance^6^, have reported that, *Mus musculus* individuals maintained under an unpredictable feeding regime reduced their rates of energy expenditure and NST. In the same way, low-energy diets lead to a reduction in NST in *Microtus brandtii* and *Meriones unguiculatus*^61,62^. Interestingly, the reallocation described here could depend on a genetic background even within intra-specific variability. In consequence, the magnitude of the responses presented here might not be universal for all rodents, considering that it has been documented that there are differences in the thermal and behavioral responses to caloric restriction between inbred mouse strains^63^.

### Summary and future perspectives

In summary, our results support the allocation model, which indicates commitments between metabolic rates and exploratory behavior, in a caloric restricted environment. Specifically, we identified the role of thermogenesis as a pivotal budget item, in the modulation of reallocation of energy between behavior and BMR. We conclude that BAT and liver play a key role in the development of paradoxical responses to dietary availability constraints.

We propose that the physiological changes observed in *Mus musculus* in a caloric restricted environment could be a common phenomenon in rodents, and likely explain some of their invasive capacities in a strategy of energy allocation to favor an exploratory behavior. Even though the most of these responses have been studied in laboratory animals, we suggest that our results could be extrapolated to nature, under certain conditions. In fact, many studies indicate that laboratory rodents (rats and mice) show an increase in their activity under food deprivation, which is proportional to the severity of food restriction^64^. Because animals often face nutritional bottlenecks^65,66^, it is not surprising that under these circumstances, the physiological and behavioral adjustments observed in the current study could occur in wild life.

Additionally, further studies about paradoxical response to caloric restriction should consider including the genetic and epigenetic contributions that drive intraspecific differences in response to food availability. Moreover, there could be an important contribution extending this analysis to several strains of *Mus musculus* or in mice under metabolic programming. Finally, a major remaining challenge in the field is how to combine these studies to changes in the expression profile of genes related to metabolism, heat production and behavior.

## Methods

### Animal acclimation

Forty-four adult males (3 months old) of the BALB/c strain of *Mus musculus* were obtained from the central animal housing facilities at the Faculty of Sciences, University of Chile. All animal procedures were in accordance with the Chilean legislation and were approved by Institutional Animal Care and Use Committees at the Universidad de Chile and Comisión Nacional de Investigación Científica y Tecnológica (CONICYT).

We design an experiment following^18^. Specifically, mice were separated into four groups of 11 animals each, considering no siblings in the same group. They were housed individually in mesh-floor cages without wood flakes, and kept at 25° ± 1 °C, in a LD = 12:12 cycle, with water *ad libitum*. Mice had no access to their feces or to the small pieces of food (<5 mm^3^). Before initiating the experimental treatments, we measured the maximum food intake in a separate group of 10 males by locating animals individually inside of cages without wood flakes and with wastage of food. From those analyses, we recorded that food intake reaches a maximal value of ~70 g per week (Table 2). Based on these results, four experimental groups were acclimated during 20 days under the following conditions: (1) continual *ad libitum* food (C-100 treatment), with 10 g of dry food per day; these individuals could eat up to 70 g per week; (2) continual feeding with restriction at 60%, with 6 g of dry food per day (C-60 treatment); these individuals could eat up to 42 g per week; (3) stochastic alimentation at 100% (S-100 treatment), with three random days of alimentation with 23 g; these individuals could eat near to 70 g per week. Finally (4), stochastic alimentation with restriction at 60% (S-60 treatment) with 3 days of alimentation with 14 g; these individuals could eat near to 42 g per week. All groups were fed with dried pellets of a commercial food (Prolab RMH 3000, Labdiet, USA).

**Table 2:** Summary of food provision and food intake values.

Treatment	Food provision (g/day)	Food intake range (g)	Theoretical requirements range (g)
Continuous alimentation with 60% of the *ad libitum* offer	6	5.40–4.02	4.96–4.10
Stochastic alimentation with 60% of the *ad libitum* offer	14*	10.5–5.28	5.08–3.83
Continuous alimentation *ad libitum*	10	7.37–3.69	5.00–3.86
Stochastic alimentation *ad libitum*	23*	10.74–4.60	4.88–3.83

Theoretical requirements were determined from metabolic estimates by means of the body mass (as indicated in^80^) and assuming a metabolizable energy of commercial pellet of 3.16 kcal/g (Prolab RMH 3000). Asterisk (*) indicate groups that fed three days by week, randomly assigned.

### Body temperature

In order to analyze the surface temperature of the animals during the whole acclimatization, a 3 g temperature data-logger (I-Buttons model DS1921L, Dallas Semi-conductors, USA) was installed on the back of each individual^8,67^. Sensors allowed us to register the skin temperature of the animals every 30 minutes for 20 days. After the animals were sacrificed (see below), the sensors were extracted and analyzed with the OneWireViewer software (0.3.17.44). Based on this information, the minimum, maximum and average body temperatures were calculated.

### Metabolic measurements

Metabolic rates were estimated via oxygen consumption rate (VO_2_) using a FoxBox respirometer (Sable Systems, Las Vegas, NV), following a modified protocol of  ^24^. Oxygen consumption was measured in 6-hours fasted animals using an illuminated chamber (0.75 L) located in a controlled temperature cabinet (Sable Systems, Henderson, Nevada) and kept at a constant ambient temperature within the thermoneutral zone for these species (Ta = 30 ± 0.5 °C). During measurement of BMR, we passed both incurrent and excurrent gas streams (500 mL min^−1^) through columns of Drierite and Baralyme to remove H_2_O and CO_2_. Output from the oxygen analyzer (%) was digitalized using a Universal Interface II (Sable Systems) and recorded using EXPEDATA data acquisition software (Sable Systems). Our sampling interval was 5 s. All measurements were made during the resting phase (09:00 and 15:00 h). Oxygen consumption was calculated according to^68^ and BMR was estimated as the average lowest oxygen consumption, under a stable register form 300 samples (25 min).

### Non-shivering thermogenesis

In order to analyze the thermogenic capacities independent of muscular activity, the NST was measured by the administration of norepinephrine (norepinephrine bitartrate, Sigma-Aldrich) and the subsequent oxygen consumption record. For this, the metabolic rate was recorded for one hour at 25 °C in an open respirometry system, and then approximately 0.02 mg of norepinephrine was administered in a volume of 100 μL^69^, by means of a suprascapular subcutaneous injection to each individual. Finally, the oxygen consumption after the injection was recorded for one hour at 25 °C^70^.

### Home-cage behavior

Home-cage behavior (i.e., cages where the individuals were acclimatized) was analyzed at the end of the acclimatization. In short, the rodents were filmed for 10 minutes, in the activity phase (21:00 to 2:00) in absolute darkness. The records were made using an infrared camera (Wanscam HW0026) located 1.5 meters above the cages on an articulated support. The camera was connected to a personal computer, arranged in an adjoining room, where filming was recorded. Subsequently, the behaviors present in the filming were analyzed through BORIS software (Behavioral Observation Research Interactive Software)^71^, quantifying the time allocated to four categories: general activity, food, hygiene and rest behaviors^72^.

### Exploratory behavior

The exploratory behavior was assessed through an open field test^36^ both at the beginning and end of the acclimation period. To this end, a square acrylic box (thickness of 2 mm) of white color was used, with an internal surface of 1 m^2^ and a height of 30 cm. These measurements were recorded within the activity phase of the species (21:00 to 02:00) as briefly described: at the beginning of the measurement the rodents were placed in the center of the field and for 10 minutes they were filmed in darkness using an infrared camera (Wanscam HW0026) located 2 meters above the exploration sand, in an articulated support. The camera was connected to a personal computer, arranged in an adjoining room where filming was recorded. Between each session the soil surface was completely cleaned with 70% ethanol. Running or walking are considered as an exploration index^73^, therefore, as a proxy of exploration or trajectory, we measured the number of squares crossed in the open field (internal surface of 1 m^2^, was digitally sectioned in a grid of 25 squares of 0.2 × 0.2 m). Then, we measured delta of trajectory in the exploration test (i.e., Δ_t_ = number of squares crossed before acclimatization - number of squares crossed after acclimatization). Δt was calculated to analyze the change in the magnitude of the displacement of mice in OFT, considering that food deprivation treatments could increase the exploratory behavior of individuals. Video analysis was performed with Phobos software^74^.

After all measurements, animals were sacrificed by cervical dislocation, then liver and fat deposits were weighed (±0.001 g; Analytical Balance, AUX Series, Shimadzu Scientific Instruments) i.e., brown adipose tissue (BAT), epididymal, and inguinal fat pads. Besides, immediately after its sacrifice, the liver and the musculature present in the hind legs (*biceps femoris, gracilis, semitendinosus, rectus femoris, anterior tibialis and vastus lateralis*) were dissected on ice. The tissues were stored at −80 °C, pending the completion of enzymatic assays.

### Metabolic enzymes

The enzymatic activity determinations were carried out using hind legs muscles and liver tissue homogenates. Samples were homogenized on ice (1:10 w/v) in phosphate buffer 0.1 M supplemented with 2 mM EDTA (pH 7.3) using an Ultra Turrax homogenizer (20000 rpm). The samples were then sonicated at 130 W using an Ultrasonic Processor VCX 130 on ice 14 times in 20-second cycles and 10-second interval between cycles. Homogenates were then centrifuged at 15,000 rpm for 15 min at 4 °C to obtain a post mitochondrial fraction. The supernatant was transferred into a new tube, to avoid transferring the upper lipid layer present in the homogenates. Protein concentration was determined using the method by^75^ with bovine serum albumin as the standard. We measured the activity of two mitochondrial enzymes: cytochrome c oxidase (COX; E.C. 1.9.3.1) and citrate synthase (CS; E.C. 4.1.3.7). An increase in the activity of these enzymes likely reflects changes in both the functional properties and the density of mitochondria. The COX activity was quantified using a microplate spectrophotometric method slightly modified from that reported by^76^. In brief, enzyme activity was determined in a reaction mixture containing 10 mM Tris-HCl (pH 7), 120 mM KCl, 250 mM sucrose, and cytochrome c reduced with dithiothreitol in a final volume of 0.2 ml. The decrease in extinction at 550 nm was monitored in a Thermo Scientific Multiskan GO UV/VIS spectrophotometer at 25 °C. Enzyme activity was calculated using an extinction coefficient of 21.84 mM^−1^ cm^−1^ at 550 nm for cytochrome-c. The CS activity was measured according to^77^ with slight modifications. The enzyme assay medium contained 10 mM Tris-HCl (pH 8.0), 10 mM 5,5′dithiobis- (2 nitrobenzoic acid), 30 mM acetyl Coenzyme A (acetyl CoA) and 10 mM oxaloacetic acid (OAA) in a final volume of 0.2 ml; these reagents were omitted in controls. Citrate synthase catalyzes the reaction between acetyl CoA and OAA to form citric acid. The increase in extinction at 412 nm was measured in a Thermo Scientific Multiskan GO at 25 °C. Enzyme activity was calculated using an extinction coefficient of 13.6 mM^−1^cm^−1^ at 412 nm. All enzyme activities are reported as specific activity per gram of protein (µmol min^−1^ mg protein^−1^).

### Statistical analysis

The averages of the morphological, biochemical and behavioral variables of the different treatments were compared through one-way ANOVA and factorial ANOVA. In cases in which the variables correlated with the body mass, the residuals of each variable against body mass were used for the regression analyzes. In addition, the existence of possible associations between the variables related to rates of energy expenditure and those variables related to exploration and general activity was evaluated by means of Pearson correlations.

Repeatability analysis was performed for exploratory behavior at the beginning and end of acclimation. Intraclass correlation coefficient (ICC) was used for the analysis through a two-way mixed effects, consistency, and multiple measurement. Interpretation was as follows: <0.50, poor; between 0.50 and 0.75, fair, between 0.75 and 0.90 good; above 0.90, excellent^78^.

We used Generalized Linear Modeling (GLM) to test the influence of periodicity, caloric intake (as dummy variables), and the interaction between these factors and metabolic rate, temperature and body/organ mass on the exploratory and home-cage behavior. All non-significant interaction effects (p > 0.05) in the GLM analyses were removed to obtain the best-fitted model in each case. We selected three models by behavioral variable, which best fitted our data by selecting the model that yielded the lowest Akaike’s information criterion, AIC^79^. All statistical analyses were performed using the R statistical environment version 3.6.1.
